# Vaccine Induced Immune Thrombotic Thrombocytopenia Causing a Severe Form of Cerebral Venous Thrombosis With High Fatality Rate: A Case Series

**DOI:** 10.3389/fneur.2021.721146

**Published:** 2021-07-30

**Authors:** Markus Wiedmann, Thor Skattør, Arne Stray-Pedersen, Luis Romundstad, Ellen-Ann Antal, Pål Bache Marthinsen, Ingvild Hausberg Sørvoll, Siw Leiknes Ernstsen, Christian G. Lund, Pål Andre Holme, Tonje Okkenhaug Johansen, Cathrine Brunborg, Anne Hege Aamodt, Nina Haagenrud Schultz, Karolina Skagen, Mona Skjelland

**Affiliations:** ^1^Department of Neurosurgery, Oslo University Hospital, Oslo, Norway; ^2^Department of Radiology and Nuclear Medicine, Oslo University Hospital, Oslo, Norway; ^3^Department of Forensic Sciences, Oslo University Hospital, Oslo, Norway; ^4^Faculty of Medicine, Institute of Clinical Medicine, University in Oslo, Oslo, Norway; ^5^Department of Anesthesiology and Intensive Care Medicine, Oslo University Hospital, Oslo, Norway; ^6^Department of Pathology, Oslo University Hospital, Oslo, Norway; ^7^Norwegian National Unit for Platelet Immunology at University Hospital of North Norway, Tromsø, Norway; ^8^Department of Neurology, Oslo University Hospital, Oslo, Norway; ^9^Department of Haematology, Oslo University Hospital, Oslo, Norway; ^10^Department of Neurosurgery, St. Olav's University Hospital, Trondheim, Norway; ^11^Oslo Centre for Biostatistics and Epidemiology, Research Support Services, Oslo University Hospital, Oslo, Norway; ^12^Department of Haematology, Akershus University Hospital, Lillestrøm, Norway

**Keywords:** COVID-19, SARS-CoV-2 virus, sinus vein thrombosis, thrombocytopenia, cerebral venous thrombosis, complication, COVID-19 vaccine AstraZeneca, ChAdOx1 nCoV-19

## Abstract

During a 2-week period, we have encountered five cases presenting with the combination of cerebral venous thrombosis (CVT), intracerebral hemorrhage and thrombocytopenia. A clinical hallmark was the rapid and severe progression of disease in spite of maximum treatment efforts, resulting in fatal outcome in for 4 out of 5 patients. All cases had received ChAdOx1 nCov-19 vaccine 1–2 weeks earlier and developed a characteristic syndrome thereafter. The rapid progressive clinical course and high fatality rate of CVT in combination with thrombocytopenia in such a cluster and in otherwise healthy adults is a recent phenomenon. Cerebral autopsy findings were those of venous hemorrhagic infarctions and thrombi in dural venous sinuses, including thrombus material apparently rich in thrombocytes, leukocytes and fibrin. Vessel walls were free of inflammation. Extra-cerebral manifestations included leech-like thrombi in large veins, fibrin clots in small venules and scattered hemorrhages on skin and membranes. CVT with thrombocytopenia after adenovirus vectored COVID-19 vaccination is a new clinical syndrome that needs to be recognized by clinicians, is challenging to treat and seems associated with a high mortality rate.

## Introduction

In Norway, a country with a population of 5.4 million, the SARS-CoV-2 vaccination program started December 27th 2020. As of 18th of April, 24% of the population had received the first vaccine dose, and 6.8% were fully vaccinated (1). The vaccination program initially started with the BNT162b2 vaccine (Comirnaty, BioNtech/Pfizer), followed by LNP-encapsuled mRNA vaccine (Moderna) in mid-January and ChAdOx1 nCoV-19 vaccine (Vaxzevria; COVID-19 vaccine AstraZeneca) in the second week of February 2021. The ChAdOx1 nCoV-19 vaccine was mainly distributed to health care workers <65 years and a total of 132 488 first doses had been administered in a 5-week period until halted by the health authorities on March 11th. Within 2 weeks, five cases of severe cerebral venous thrombosis (CVT), associated with intra-cerebral hemorrhage and thrombocytopenia and one case with splanchnic vein thrombosis and thrombocytopenia were encountered in previously healthy health care workers after having received ChAdOx1 CoV-19 vaccine.

This vaccine-induced immune syndrome of severe thrombosis, high levels of antibodies to platelet factor 4–polyanion complexes and thrombocytopenia was recently reported by our group and the term VITT was proposed (1). Including our previously reported cohort of VITT patients, we further characterize the malignant clinical entity of CVT and thrombocytopenia caused by this syndrome, with focus on neurological symptoms, radiology and pathology findings.

## Methods

### Ethics

Written informed consent for publication was obtained from patients or their next of kin.

### Pathology

Three fatalities (case 2, 3 and 5) were subject to forensic post-mortem examination including full body CT and supplementary analyses including histology, neuropathological examination and whole exome sequencing. Case 1 was examined by hospital autopsy upon request of the clinicians and with permission from next-of-kin.

### Laboratory Tests

Anti-PF4/polyanion antibody testing of patient serum was performed by ELISA (LIFECODES PF4 IgG, Immucor) including a heparin inhibition test, as described previously (2). The ability of patient serum to functionally activate and aggregate platelets was tested by the heparin-induced multiple-electrode aggregometry (HIMEA) on a Multiplate analyzer (Dynabyte Medical) under saline conditions (2).

### Data Analysis

Vaccination data were obtained from the national vaccination registry at the National Institute of Health. Standard statistical methods were used to describe the data, including calculation of relative risk and 2 × 2 tables. Statistical analyses were performed by using Stata (Version 16 SE; StataCorp LLC) and OpenEpi: Open Source Epidemiologic Statistics for Public Health, Version. www.OpenEpi.com, updated 2013/04/06, accessed 2021/04/19.

## Results

In the period from 8.2.2021 to 11.3.2021, a total of 132 488 individuals received the first dose of ChAdOx1 nCoV-19 vaccine. 77% of the vaccinated were women and 68% of the vaccinated were between 30 and 59 years of age (Supplementary Table I). In total, 6 patients (5 women and one man) developed what was later named vaccine induced immune thrombotic thrombocytopenia (VITT) (2). The incidence of VITT in Norway to date is 1 per 22 000 vaccinated with ChAdOx1 nCoV-19. VITT was not more common in women than in men (RR = 1.47 95% CI 0.17, 12.6) and was not more common in the age group 20–39 compared to 40–69 (RR = 3.27 95% CI 0.60, 17.9).

Five of those VITT patients presented with a characteristic clinical syndrome of cerebral venous thrombosis, parenchymal hemorrhage and severe thrombocytopenia, of whom four cases were described in detail in a previous case report (2). The fifth case (Case 1) was diagnosed with VITT post mortem as she was the very first case in Norway and died before the syndrome was recognized.

Basic clinical and biochemical characteristics of the five patients are presented in Supplementary Tables I, II and 4 out of 5 patients have been reported previously (2). All patients were female and 34 to 54 years of age. They presented with combined moderate to severe thrombocytopenia and CVT, 7–10 days after vaccination with ChAdOx1 nCoV-19. None had received heparin prior to symptom onset and none tested positive for SARS-CoV-2 virus or had previous Covid-19 infection. All patients except one had a fatal outcome.

### Case Descriptions

#### Patient 1

**Figure 1:**
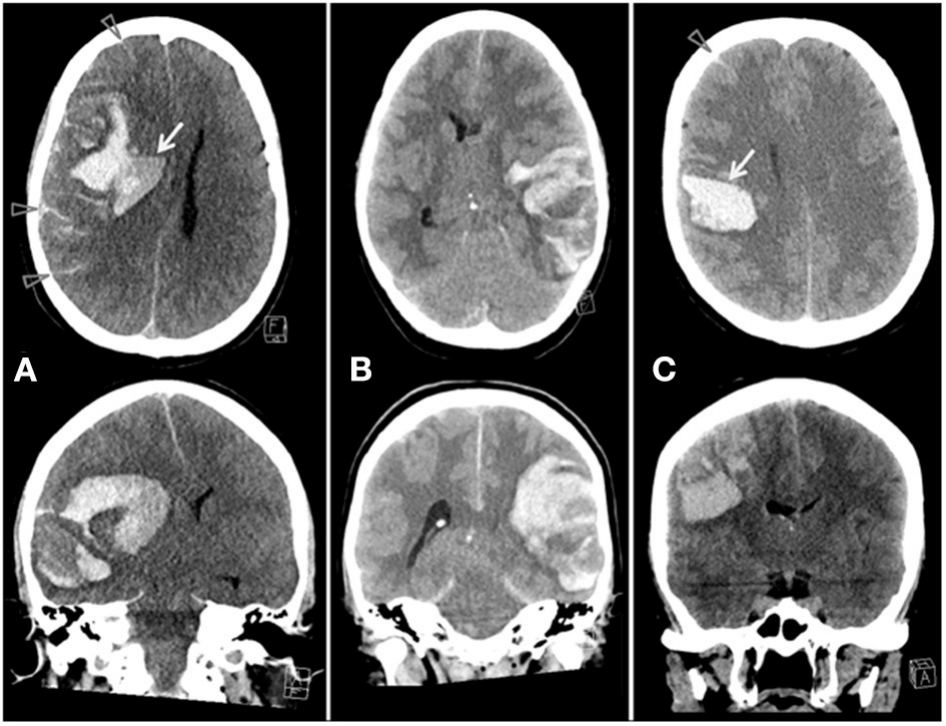
Axial and coronal CT from patient 1 **(A)**, 2 **(B)** and 5 **(C)**. These patients presented with large parenchymal hemorrhages. Note the fluid-fluid levels [arrows, **(A,C)**], the subarachnoid hemorrhage [arrowheads, **(A,C)**] and the heterogeneous appearance of all hematomas.

This patient was a 34 year-old previously healthy woman who developed headaches on day 7 after vaccination with ChAdOx1 nCoV-19. Due to increasing symptoms, including intense headaches, left-sided limb weakness and dysarthria, she was admitted to hospital. On examination, she was drowsy, dysphasic and had a left sided hemiparalysis with vertical gaze deviation. Cerebral CT demonstrated a large right-sided parenchymal and subarachnoid hemorrhage. She presented with a Glasgow Coma Score (GCS) of 3 and bilateral pupillary dilatation. Repeat cerebral CT demonstrated increased parenchymal hemorrhage, mass effect and herniation (Figure 1). This patient died the day after hospital admission.

Autopsy demonstrated an edematous brain with sparse subarachnoid hemorrhage and a large hemorrhagic infarction in the right hemisphere. Thrombi were present in both transverse sinuses. Scattered petechial and flame-shaped hemorrhages were observed on the skin, peritoneal membranes and mucosal surfaces. No thrombi were observed in peripheral veins or in extracerebral organs present for examination.

#### Patient 2

**Figure 2:**
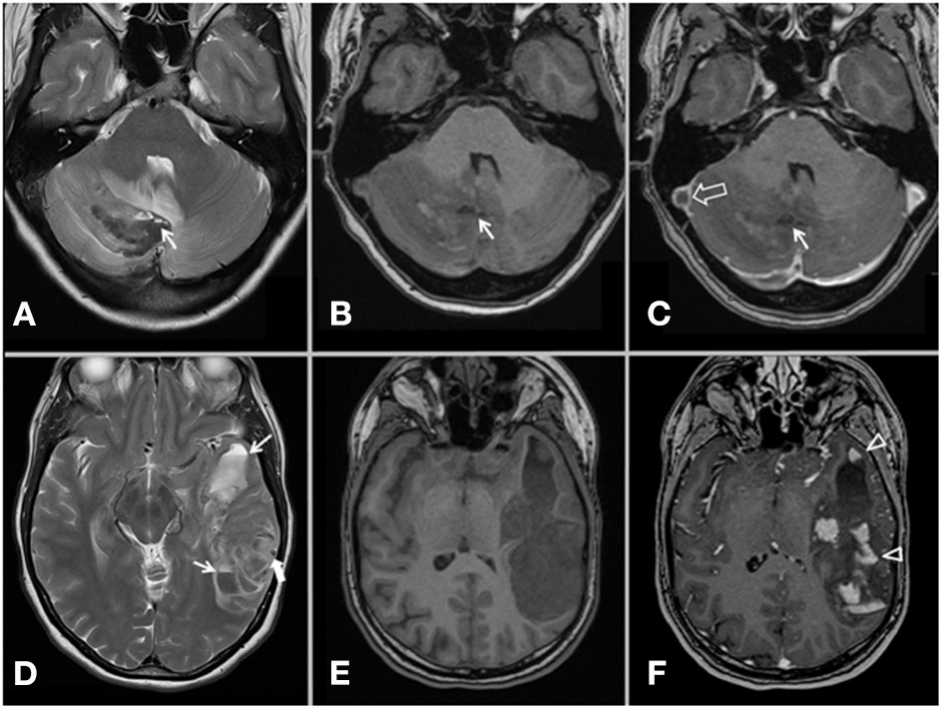
MRI of patient 4 (upper row) and patient 2 (lower row), axial T2-weighted images **(A,D)**, axial T1-weighted images **(B,E)** and contrast-enhanced axial T1-weighted images **(C,F)**. A complex signal in hematomas of venous infarctions in the right cerebellar hemisphere (patient 4) and left temporal lobe (patient 2) is seen. Multiple fluid-fluid levels (arrows) and sedimentation indicate not fully coagulated blood. Multifocal areas of high signal intensity within the hematoma represents extravasated contrast agent [arrow heads, **(F)**]. An acute thrombus in the right transverse and sigmoid sinus [open arrow, **(C)**] cause a well-defined filling defect on the contrast-enhanced T1-MPRAGE. The round lesion with low signal intensity on T2-weighted image [thick arrow, **(D)**] was tubular on consecutive slices, showed lack of contrast enhancement and is compatible with a cortical vein thrombosis.

This 42-year-old woman was admitted to hospital on day 10 after vaccination with ChAdOx1 nCoV-19 with severe headaches, nausea, vomiting, fluctuating level of consciousness and right sided hemiparesis. Initial cerebral CT with venography revealed a left sided lobar, heterogenous hemorrhage with fluid levels (Figure 1) and contrast defects in the left transverse and sigmoid sinus. MRI venography confirmed cerebral sinus vein thrombosis (CSVT) and cortical vein thrombosis (CoVT) (Figure 2).

The patient deteriorated 9 h after hospital admission and developed a left sided pupillary dilatation. A repeat CT revealed progression of mass effect, brain herniation and cortical subarachnoid hemorrhage. An emergency left-sided decompressive hemicraniectomy was performed. Intravenous methylprednisolone (1 mg/kg) daily and intravenous immunoglobulin (IVIG) (1 g/kg) for 2 days were started on day 7 after admission. Platelet counts stabilized and steadily increased reaching 680 10^9^/l on day 15 and LMWH was increased gradually to therapeutic levels (Supplementary Figure I). However, the intra-cranial situation gradually escalated with expansion of venous infarctions and secondary hemorrhages in both hemispheres and gradually increasing intra-cranial pressure (ICP). The patient died on day 15 after hospital admission.

At autopsy, a red-white clot was confirmed present in the left transverse and sigmoid sinus, as well as remnants of white clots attached to the endothelium in the sagittal sinus. Massive hemorrhagic infarction was present in the left hemisphere. In the lungs, peripheral areas with infarction were demonstrated. Microscopy confirmed multiple arteriolar thrombi in organization. In addition, small venules with intraluminal fibrin clots were present in several lung lobes and also in the myocardium.

#### Patient 3

**Figure 3:**
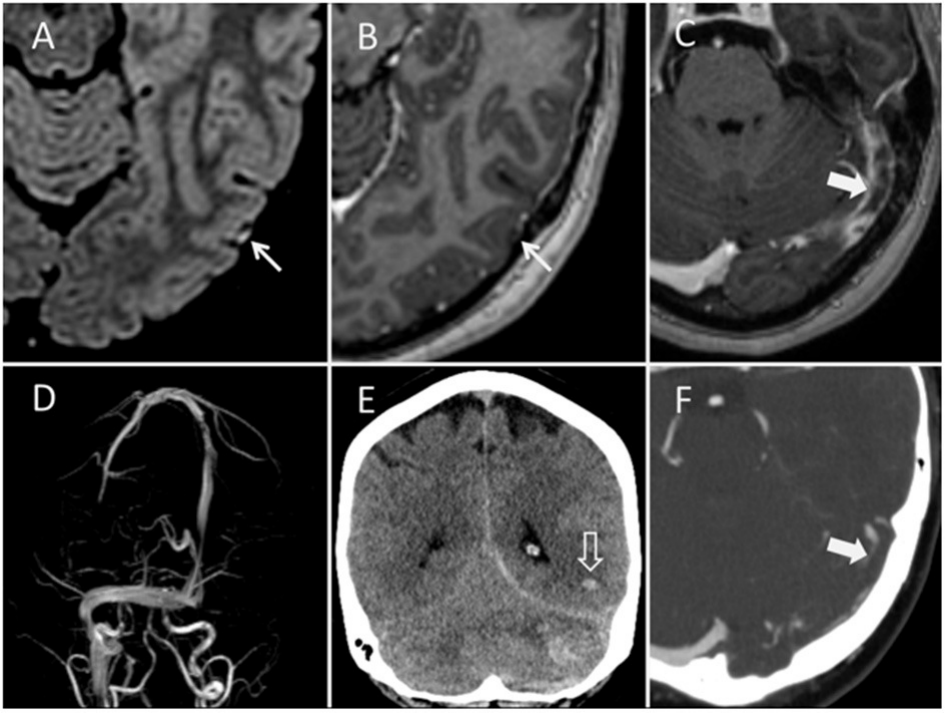
MRI, CT, and CT venography of patient 3. Thrombosis in a cortical vein (thin arrows) is possible to spot with high signal intensity on axial FLAIR **(A)** and filling defect on contrast enhanced axial T1-MPRAGE **(B)**. Corresponding temporo-occipital hemorrhage (open arrow), cerebellar hemorrhage and high attenuation from thrombus in transverse and sigmoid sinuses can be seen on coronal CT **(E)**. 3D phase-contrast MR venography demonstrates loss of flow signal in left transverse and sigmoid sinus **(D)**. The thrombus in sigmoid sinus (thick arrows) is also evident on contrast enhanced axial T1-MPRAGE **(C)** and CT venography **(F)**.

This 37-year-old woman presented to hospital on day 8 after vaccination with ChAdOx1 nCoV-19, with a 2-day history of headaches, fever, transient numbness in the right foot and right sided visual disturbance. Cerebral CT and MRI demonstrated CSVT in the left transverse and sigmoid sinus, and left occipital CoVT (Figure 3).

The patient deteriorated clinically the day after admission with increasing headaches and visual disturbances. A repeat cerebral CT demonstrated temporo-occipital, subarachnoid and left cerebellar hemorrhage, with significant mass effect. Rapid clinical deterioration with decreasing level of consciousness (GCS 15 to 3), alongside a left sided pupillary dilatation were observed. Urgent suboccipital craniectomy was performed and cerebellar herniation encountered during surgery. A post-operative CT demonstrated cerebellar edema and hemorrhage with brain stem compression and cerebellar herniation through the craniectomy. The patient died on day 11 after vaccination.

Neuropathological examination revealed a large hemorrhagic infarction in the left cerebral hemisphere, extensive hemorrhagic changes in the cerebellum, as well as focal white substance hemorrhages in the cerebral hemispheres and in the brainstem. Thrombi were present in the left transverse and sigmoid sinuses. Scattered small hemorrhages were observed on the skin and peritoneal membranes.

#### Patient 4

This 39-year-old woman presented to emergency clinic on day 7 post ChAdOx1 nCoV-19 vaccination with abdominal pain and headaches. Mild isolated thrombocytopenia (119 × 10^9^/l) was diagnosed, abdominal ultrasound was normal and she was discharged, as she was clinically improving. Due to persisting headaches, she re-presented to emergency clinic 2 days later. On admission, she was fully alert and had no neurological deficits. A cerebral CT, however, demonstrated a small cerebellar hemorrhage. MR venography showed CSVT in the inferior sagittal sinus, vein of Galen and straight, right transverse and sigmoid sinuses (Figure 2). CT pulmonary angiogram showed bilateral segmental pulmonary emboli and abdominal CT demonstrated thrombosis in uterine veins. She recovered slowly after treatment with IVIG, steroids and LMWH and was finally discharged. MRI with venography before discharge demonstrated recanalization of the sagittal and straight sinus, and partial recanalization of transverse and sigmoid sinus. Anticoagulation with warfarin was continued.

#### Patient 5

This 54-year-old woman felt unwell and reported numbness of her left-sided limbs 6 days post vaccination. The next day, her symptoms had progressed and she woke up with headaches, nausea and left sided weakness. On admission, she was somnolent with a GCS of 14 and several skin bruises were noted, alongside with left sided hemiparalysis and facial nerve palsy. Cerebral CT revealed right hemispheric parenchymal hemorrhage (Figure 1). Repeat cerebral imaging demonstrated progression of venous infarction and parenchymal and subarachnoid hemorrhage with massive CSVT in nearly all major venous sinuses. Methylprednisolone (1 mg/kg) and IVIG (1 g/kg) for 2 days were administered and endovascular mechanical venous thrombectomy was performed. Venous recanalization was achieved, but a dilated right pupil developed at the end of the procedure. A CT scan revealed further progression of parenchymal hemorrhage. Decompressive hemicraniectomy was performed. Despite maximum treatment efforts in intensive care unit, ICP continued to increase and she died 2 days later.

**Figure 4:**
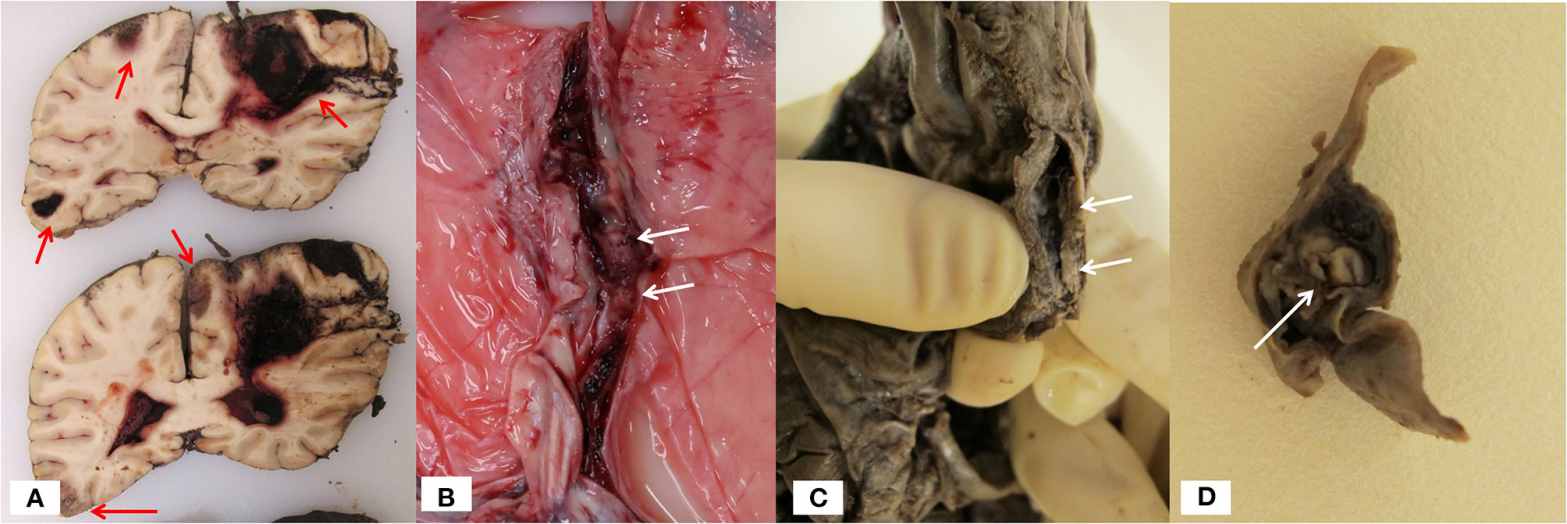
Autopsy findings in patient 5: **(A)** Coronal section of the formalin-fixed brain. Extensive hemorrhagic infarction (red arrows) in both hemispheres and major hemorrhage with communication to the brain surface and ventricular system. **(B)** Posterior sagittal sinus with a white thrombus. **(C)** Formalin fixed dura. Sagittal sinus with thrombus formation (arrow). **(D)** Cross-section of formalin fixed right transverse sinus with white thrombus (arrow).

Neuropathological examination demonstrated a white clot in the posterior sagittal sinus and both transverse sinuses (Figure 4). Massive hemorrhagic venous infarction was confirmed in the right parietal lobe and bilateral hemorrhagic infarctions in multiple cortical areas (Figure 4). There were multiple extra-cerebral manifestations of coagulation disturbance, with leech-like white thrombi in the inferior vena cava, left subclavian trunk, right inter-atrial septum, and both portal and hepatic veins (Supplementary Figure II). Microscopically, these extra-cerebral thrombi were rich in platelets, fibrin and leukocytes with abundance of monocytes, and were attached to the endothelium, but without signs of organization. In the spleen, subcapsular hemorrhages were present as well as multiple intralobular arterioles with fibrinoid necrosis. No pre-existing pathology in organs or bone marrow was detected.

## Discussion

We describe five patients presenting with rapid progressive neurological symptoms, CVT with intracerebral hemorrhage and thrombocytopenia, occurring 7–10 days after vaccination with ChAdOx1 nCoV-19. This characteristic clinical picture of CVT in combination with thrombocytopenia is the most prominent presentation of VITT, a recently described complication (1, 2) after vaccination with ChAdOx1 nCoV-19.

VITT seems to occur about 1–2 weeks after vaccination with ChAdOx1 nCoV-19 and describes the rapid development of thrombosis at unusual sites (e.g., cerebral and splanchnic veins), and has similarities to *autoimmune* heparin induced thrombocytopenia (HIT), that may occur without previous heparin exposure, in contrary to classic HIT in patients treated with heparin (3).

The diagnosis of CVT in context with COVID-19 vaccination can be challenging as the symptoms initially can be discrete and unspecific. Headache, the most common initial symptom of CVT, is also commonly encountered after COVID-19 vaccination and does therefore not rise any red flags. However, the flu-like symptoms after COVID-19 vaccination mostly occur in the first days, in contrary to the increasing symptoms about a week after vaccination, as experienced by our patients. Petechiae and ecchymosis were observed in 4 of 5 patients and may represent an early clinical finding. The presence of neurological symptoms after ChAdOx1 nCoV-19 vaccine should prompt further investigations, including platelet count, ELISA testing for platelet factor 4 (PF4) –polyanion antibodies and cerebral imaging with venography. However, on admission of our first cases, the clinical picture was not well-understood.

Parenchymal hemorrhage occurred in all cases, and occurs normally in 30–40% of patients with CVT (3). The parenchymal hemorrhages appeared heterogeneously with variable signal (MRI) and attenuation (CT), and fluid-fluid levels, sedimentation and multifocal leak of contrast media were found (Figures 1, 2). Three of our patients had hematomas in locations not usually associated with CSVT, two cerebellar and one centered around the sylvian fissure. Four patients also had subarachnoid hemorrhage, a rare presentation of CVT (4, 5). CoVT was diagnosed in three of the fatal cases and also suspected in the fourth fatal case (case 1), based on the location of hemorrhages, although venography had not been performed. In general, CSVT with concomitant CoVT is associated with severe clinical manifestations and poor outcome (6). Thrombosis of small veins, thrombocytopenia and coagulopathy may explain the characteristics and atypical radiological findings of hemorrhage secondary to CVT in context of VITT.

Blood clots generated in the arterial circulation are generally rich in platelets and appear white at autopsy. Clots from the venous circulation are rich in fibrin and erythrocytes, with a more red/brownish appearance and are typically found in CVT. In light of the unusual hematological process related to the thrombus formation in our five patients and macroscopic appearance of some of the thrombus material, one may question whether the thrombi present in our cases may differ from those more commonly formed in classical arterial and venous thromboembolic processes. Experimental studies have demonstrated significant changes in clot microstructure in HIT (7). Ultra-large complexes between heparin polysaccharides and PF4 activate platelets and the immune system, causing release of tissue factor and activation of the coagulation cascade with subsequent generation of thrombin (7). Tissue factor is widely present in brain tissue and release into the capillary bed may be further exacerbated by changes in the microcirculation after early thrombus formation. The predilection of CVT in VITT is peculiar and the pathophysiological mechanisms not well-understood. The cerebral venous system contains particular structures and physiological properties (8), that may be more prone to thrombosis in VITT, but this needs to be elucidated further.

The combination of CVT and cerebral hemorrhage, together with thrombocytopenia is exceedingly rare and confronts the clinician with therapeutic decisions to (a) control cerebral hemorrhage, (b) re-institute platelet function and (c) efficiently lyse thrombi obstructing venous outflow.

Cornerstone in CVT treatment in the acute phase is anti-coagulant therapy with heparin or preferentially LMWH in spite of cerebral hemorrhage (9). Decompressive surgery may be considered for patients with parenchymal lesions, edema and impending herniation (9). According to recent publications, anticoagulation with DOACS (direct acting anti-coagulants) is comparable to warfarin in some cases of CVT, but not in the acute phase with parenchymal hematoma or in a setting of major neurosurgical intervention (10, 11). Severe thrombocytopenia further increased the risk of increasing parenchymal hemorrhage in our patients and platelet transfusions were therefore indicated. Based on few cases of autoimmune HIT and a possible theoretical aggravation of the situation caused by heparin, an alternative anticoagulation strategy has been recently suggested in the management of VITT (12, 13). Therefore, one could also argue for a different treatment strategy in our patients. However, anticoagulation in patients with severe thrombosis combined with thrombocytopenia and major bleeding is challenging. In severe cerebral hemorrhage, anticoagulants need to administered cautiously, should have a relatively short half-life and be reversible. In this setting, DOACs and fondaparinux are not considered safe treatment options and are generally not indicated in cases with CVT, severe hemorrhage and in context of neurosurgical intervention. Platelet counts were increasing after initiation of IVIG and steroid treatment and continued to increase in spite of therapeutic LMWH treatment. Also, in HIMEA functional testing, platelets in serum from patients 1, 3, 4 and 5 were clearly activated in the absence of added heparin (2). This is in concordance with the report by Scully et al., where heparin did not negatively influence the treatment of 22 cases with VITT (13). Giving clear advice in the treatment of VITT in the setting of CVT combined with cerebral hemorrhage is therefore difficult and no clear conclusion can yet be drawn. At least, there seems to be consensus that immune modulating treatment is indicated and should be administered promptly when suspecting VITT (2, 12).

Outcome in our case series was extremely poor and differs significantly from previous reports on CVT, including large proportions of initially severely ill patients (14). In a systematic review of selected patients for venous thrombectomy, 60% initially presented with intracerebral hemorrhage and 47% with significantly reduced level of consciousness. Yet, good outcome was achieved in 84% and mortality was 12% (14). The 30-day case fatality rate in the large International Study on Cerebral Vein and Sinus Thrombosis was 3.4% only (15). This is in clear contrast to our case series with an 80% case fatality rate in previously healthy patients and in keeping with the bad outcome reported for CVT in VITT by others (12, 13).

We describe the clinical, radiological and pathological findings of severe thrombocytopenic, hemorrhagic cerebral venous thrombosis in VITT after ChAdOx1 nCoV-19 vaccine. Although an association with ChAdOx1 nCoV-19 vaccine seems obvious, the exact cause of this immune reaction is not yet clear. Outcome in our cohort was extremely poor and a contributing factor may have been the delayed recognition of this syndrome. A high level of suspicion is therefore warranted in individuals, vaccinated with ChAdOx1 nCoV-19, who develop symptoms such as increasing headaches, skin hemorrhages, signs of neurological deficits and thrombocytopenia and early investigation for VITT is indicated.

## Data Availability Statement

The original contributions presented in the study are included in the article/Supplementary Material, further inquiries can be directed to the corresponding authors.

## Ethics Statement

Ethical review and approval was not required for the study on human participants in accordance with the local legislation and institutional requirements. The patients/participants provided their written informed consent to participate in this study.

## Author Contributions

MW, TS, AS-P, LR, IS, PH, TJ, AA, NS, KS, and MS contributed to data collection and conception of the study. NS, LR, MW, and MS organized the patient data. TS, PM, AS-P, NS, and MS concepted the figures. CB performed the statistical analysis. MW wrote the first draft of the manuscript. TS, PM, NS, IS, SL, AS-P, KS, and MS wrote sections of the manuscript. All authors contributed to the article and approved the submitted version.

## Conflict of Interest

MW research grants from the South-Eastern Norway Regional Health Authority (grant number 2014060), ownership of stock Biontech/Pfizer. IS reports that her spouse is the CFO in ArcticZymes Technologies. CL personal fees from Bristol Myers Squibb. PH personal fees from Takeda, grants and personal fees from SOBI, grants and personal fees from Bayer, grants and personal fees from Pfizer, personal fees from Roche, personal fees from Octapharma, personal fees from NovoNordisk, personal fees from CSL, personal fees from BMS. AA personal fees from Bayer, personal fees from Boehringer Ingelheim, personal fees from Roche, personal fees from Allergan, personal fees from Novartis, personal fees from Teva. NS personal fees from BMS/Pfizer, personal fees from Bayer. KS personal fees from Bayer. The remaining authors declare that the research was conducted in the absence of any commercial or financial relationships that could be construed as a potential conflict of interest.

## Publisher's Note

All claims expressed in this article are solely those of the authors and do not necessarily represent those of their affiliated organizations, or those of the publisher, the editors and the reviewers. Any product that may be evaluated in this article, or claim that may be made by its manufacturer, is not guaranteed or endorsed by the publisher.

## Abbreviations

SARS-CoV-2: severe acute respiratory syndrome coronavirus 2
IVIG: intravenous immunoglobulin
VITT: vaccine-induced immune thrombotic thrombocytopenia
CVT: cerebral venous thrombosis
Covid-19: Coronavirus disease 2019
CSVT: cerebral sinus vein thrombosis
CoVT: cortical vein thrombosis
LMWH: low molecular weight heparin.
