# Supplementary material for: Vaccine Induced Immune Thrombotic Thrombocytopenia Causing a Severe Form of Cerebral Venous Thrombosis With High Fatality Rate: A Case Series
Source: Front Neurol. 2021 Jul 30;12:721146. doi: 10.3389/fneur.2021.721146 (PMC8363077; doi:10.3389/fneur.2021.721146)
Supplement: Supplementary file 1 [file Data_Sheet_1.docx]

**Supplemental Material**

**Vaccine induced immune thrombotic thrombocytopenia (VITT) causing a severe form of cerebral venous thrombosis with high fatality rate: a case series**

**Table I.** Individuals vaccinated with the first dose of ChAdOx1 nCov-19 in the period 8.2.2021 to 11.3.2021 and cases diagnosed with VITT

| Age in years | 20-29 | 30-39 | 40-49 | 50-59 | 60-69 | ≥70 | Total |
| --- | --- | --- | --- | --- | --- | --- | --- |
| Women | 19246 | 20265 | 23909 | 26331 | 12349 | 275 | 102380 |
| Men | 4601 | 6130 | 6228 | 7525 | 5377 | 246 | 30108 |
| VITT | - | 4 | 1 | 1 | - | - | - |
| Total | 23847 | 26399 | 30138 | 33857 | 17726 | 521 | 132488 |

**Table II.** Patient data

|  | **Case 1*** | **Case 2** | **Case 3** | **Case 4** | **Case 5** |
| --- | --- | --- | --- | --- | --- |
| Sex | Female | Female | Female | Female | Female |
| Age in years | 34 | 42 | 37 | 39 | 54 |
| Previous diseases | Pollen allergy | Pollen allergy | Pollen allergy | - | Hypertension |
| Regular medication | Contraceptive vaginal ring | Contraceptive vaginal ring | Oral contraceptive | - | Estrogen replacement, anti-hypertensiva |
| Days from vaccination to admission | 10 | 10 | 8 | 7 | 7 |
| Skin hemorrhages | Ecchymoses and petechiae | Ecchymoses | Ecchymoses and petechiae | None | Ecchymoses |
|  |  |  |  |  |  |
| Treatment measures in ICU | S, I, MV,  osmotherapy | S, I, MV,  osmotherapy, hemi-craniectomy | S, I, MV,  osmotherapy,  suboccipital craniectomy | NA | S, I, MV,  osmotherapy, hemi-craniectomy |
| Days in the ICU | 2 | 15 | 3 | 0 | 3 |
|  |  |  |  |  |  |
| Outcome | Fatal | Fatal | Fatal | Recovering | Fatal |

*Index case

NA: not applicable

S, I, MV: Sedation, intubation, mechanical ventilation

**Table** **III** Laboratory patient data

|  | **Reference** | **Case 1** | **Case 2** | **Case 3** | **Case 4** | **Case 5** |
| --- | --- | --- | --- | --- | --- | --- |
| **Hemoglobin (g/dl)** | 11.7-15.3 | 13.6 | 12.1 | 11.4 | 12.6 | 9.6 |
| **Haptoglobin (g/l)** | 0.4-2.1 | - | 0,1 | - | - | 2.0 |
| **Lowest platelet counts measured (10^9^/l)** | 145-390 | 33 | 14 | 22 | 70 | 19 |
| **PF4/PVS ELISA IgG (OD)** | <0.4 | 2.8 | 3.5 | 3.7 | 3.8 | 2.9 |
| **PF4/PVS ELISA, Heparin inhibition (%)** | <50 | 99 | 98 | 100 | 100 | 100 |
| **Platelet aggregation with patient serum^1^** | Negative | Positive | In-concl. | Positive | Positive | Positive |
| **aPTT peak** | 22-30 | 28 | 31 | 28 | 25 | 25 |
| **INR peak** | 0.9-1.2 | 1.0 | 1.0 | 1.1 | 1.3 | 1.2 |
| **D–dimer (mg/l) peak** | <0.54 | 16.2 | >35 | >35 | 13 | >35 |
| **Fibrinogen (g/L) nadir** | 1.9-4.0 | 1.9 | 0.7 | 1.8 | 1.2 | 1.1 |
| **CRP (mg/l)** | <4 | 10 | 6.1 | 5.8 | 5.1 | 61 |
| **Protein S activity (IU/dL)** | >65 | - | 50 | 74 | 78 | 81 |
| **Protein C activity (IU/dL)** | >70 | - | 75 | 78 | 101 | 94 |
| **Anti-thrombin activity (IU/dL)** | >83 | - | 100 | 76 | 126 | 90 |
| **ADAMTS13 activity (%)** | >77% | - | 97 | 98 | - | - |
| **SARS-Cov-2 RNA^2^** | Negative | Negative | Negative | Negative | Negative | Negative |

^1^Irrespective of added heparin

^2^All patients were negative for antibodies to SARS-CoV-2 nucleocapsid protein, making infection with SARS-CoV-2 highly unlikely

Prothrombin g.20210G, Factor V Leiden mutation, anti-phospholipid anti-bodies were all negative in patients 2-5; not tested in patient 1

PF4: Platelet factor 4

aPTT: Activated partial thromboplastin time

INR: International normalized ratio

CRP: C-reactive protein

**Figure I**

Platelet counts of patients 1-5 and medical treatment measures.

IVIG: Intravenous immunoglobulin

LMWH: Low molecular weight heparin


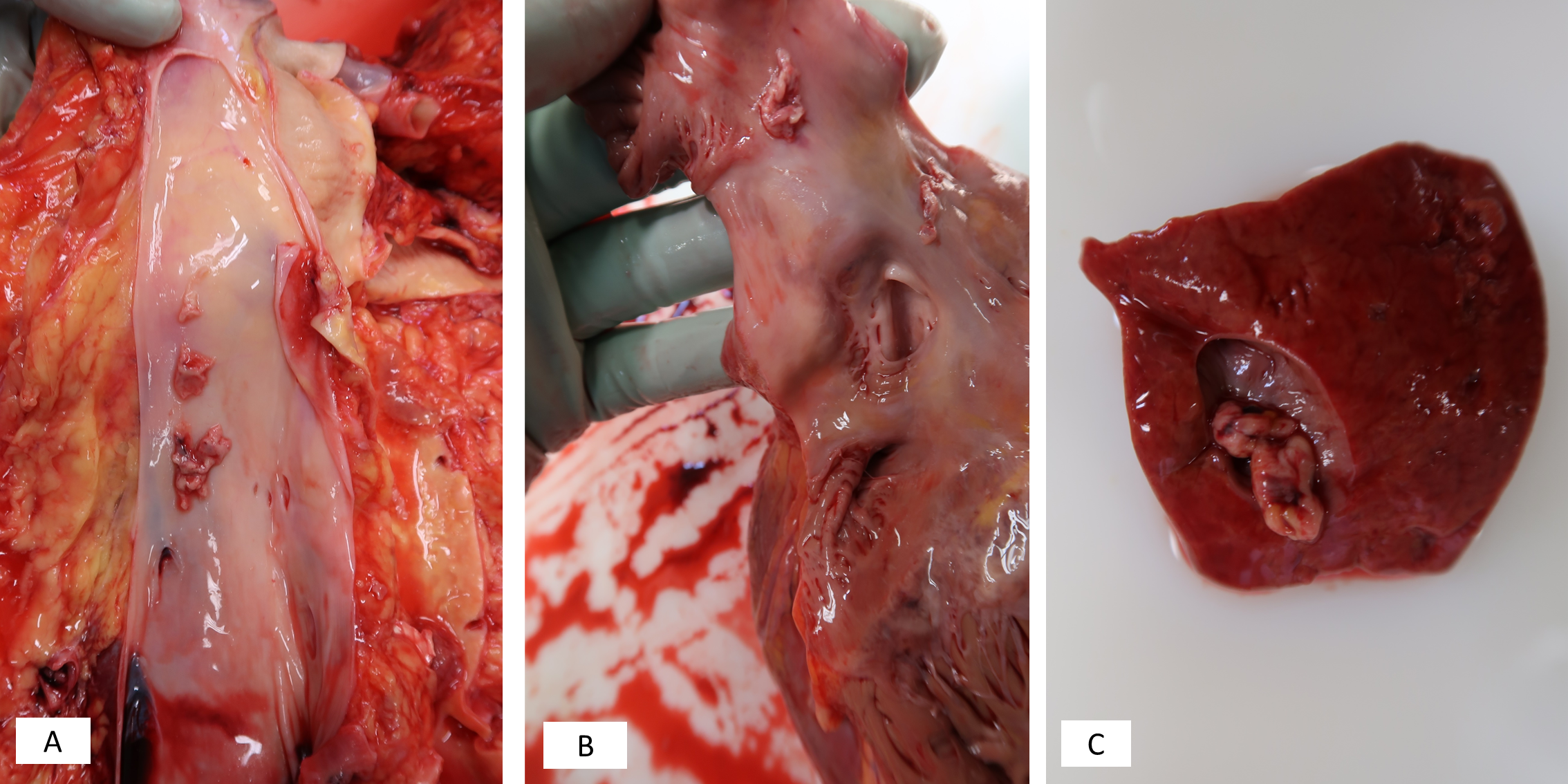


**Figure II**

Extracerebral clots demonstrated at autopsy (patient 5). A. Inferior vena cava with leech-like white clots attached to the wall. B. Right atrial wall with clots. C. Section of liver with a white clot in the hepatic vein.
